# Coinfection with* Schistosoma haematobium* and* Plasmodium falciparum* and Anaemia Severity among Pregnant Women in Munyenge, Mount Cameroon Area: A Cross-Sectional Study

**DOI:** 10.1155/2017/6173465

**Published:** 2017-01-11

**Authors:** Judith K. Anchang-Kimbi, Dillys Mansoh Elad, Gemain Taiwe Sotoing, Eric Akum Achidi

**Affiliations:** ^1^Department of Zoology and Animal Physiology, University of Buea, Buea 63, Cameroon; ^2^Department of Biochemistry and Molecular Biology, University of Buea, Buea 63, Cameroon

## Abstract

*Background*. Malaria and urogenital schistosomiasis are coendemic in Mount Cameroon Area. This study investigated the prevalence of* S. haematobium*,* P. falciparum,* and coinfections and their effect on anaemia in pregnancy.* Methods*. Pregnant women reporting for antenatal care (ANC) clinic visit in Munyenge were enrolled.* S. haematobium* and* P. falciparum* infections were determined by urine filtration and microscopy, respectively. Haemoglobin (Hb) levels were measured using haemoglobinometer. Of 250 women, 46.8%, 39.2%, and 15.2% had* S. haematobium*,* P. falciparum,* and coinfections, respectively. Schistosomes infection was higher in younger women (≤25 years) and those who bathe in and had domestic contact with stream compared with older age (>25 years) women and those who had only domestic contact with stream. Lower infection rate was associated with less water contact (≤2 times/day) compared with more water contact (>2 times/day). Compared with no sulphadoxine-pyrimethamine (SP) usage, malaria parasitaemia was less among women who used SP. Stream usage increased risk of coinfection while less water contact and SP usage decreased its risk. All coinfected cases were anaemic and coinfection accounted for 93.8% of severe anaemia.* Conclusion*. Coinfection contributes to anaemia severity. Less water contact and SP usage will reduce coinfection in pregnancy in Munyenge.

## 1. Background

Malaria parasite and helminth infections are the most prevalent parasitic diseases in developing countries and their epidemiologic coexistence is frequently observed, particularly in Africa [1]. The overlap of malaria parasite and helminth infections is influenced by high frequencies of the parasites in the same population, similar geographical distribution of parasites, shared risk factors, common transmission methods [1], and genetic and immunological predisposition [2]. Findings from epidemiological studies suggest that interactions between malaria parasite and helminth infections can be antagonistic [3, 4] or synergistic [5, 6]. Some studies have proposed an immunologic hypothesis based upon the type T cell response (Th1 or Th2) induced by each parasite [2, 7]. Synergistic T cell responses could decrease the pathological impact of the infections, whereas antagonistic T cell responses could exacerbate disease. Coinfection may have considerable health consequences leading to more severe clinical symptoms and pathology than infection with single parasite species [8, 9]. For example, coinfections of* Plasmodium falciparum* with hookworms and schistosomes tend to exacerbate hepatosplenic, anaemia, and malnutrition morbidities [8].

*Plasmodium falciparum* inflicts the greatest burden and about 90% of the populations infected with malaria live in sub-Saharan Africa. Pregnant women are particularly vulnerable to* P. falciparum* especially in first pregnancy [10] and protective interventions against malaria include intermittent preventive treatment with sulphadoxine-pyrimethamine (IPTp-SP) and use of insecticide treated bed nets (ITNs) [11]. Besides malaria, schistosomiasis is the second important parasitic disease in terms of socioeconomic and public health importance [12]. More than 90% of the roughly 200 million cases of schistosomiasis occur in Africa [13] of which approximately two-thirds are caused by* Schistosoma haematobium* [14], the etiologic agent of urogenital schistosomiasis. Schistosomiasis is endemic in rural areas where there is a lack of safe water supply, poverty, ignorance, and poor hygienic practices [15]. Forty million women of child bearing age are infected with younger and pregnant women being at greater risk of infection [16, 17]. Domestic activities such as washing clothes and fetching water in infected water expose women and children to infection [18]. In Cameroon, schistosomiasis is endemic in the northern regions [19], Centre, East, West [20], Littoral, North West, South, and Southwest Regions [21]. Urogenital schistosomiasis is endemic in Southwestern Cameroon where Barombi Kotto [22] and Munyenge [23] are identified transmission foci. While most of these studies have focused on school-age children and the community, epidemiological data on the prevalence of urogenital schistosomiasis and its burden among pregnant women living in these endemic foci is lacking.

Studies have demonstrated that schistosomiasis infection in pregnant women results in severe anaemia [16], low birth weight, and maternal mortality [24–26]. However, the aetiology of anaemia is multifactorial involving complex interaction between nutrition status, infectious disease (malaria, human immunodeficiency virus (HIV), and helminths), and other factors (sociodemographic and economic) [27]. These conditions are integrally linked and subsequently lead to adverse pregnancy outcomes [27].* S. haematobium* have been linked to placental inflammation leading to poor birth outcomes as a result of placental malfunction. Data suggest that infected women have a higher rate of spontaneous abortions and a higher risk for ectopic pregnancies [28, 29]. Conversely, studies have demonstrated the biologic plausibility that female genital schistosomiasis may make women more susceptible to HIV [30]. Praziquantel (PZQ) is recommended in pregnancy [31].

Pregnant women living in Munyenge, Mount Cameroon Area, may be exposed to coinfection with* S. haematobium* and* P. falciparum* and thus may experience anaemia severity. The specific aims of this study were to (i) determine the prevalence and intensity of* S. haematobium*,* P. falciparum*, and coinfections among pregnant women reporting for ANC clinic visit at the Munyenge Health Centre, (ii) determine the factors associated with prevalence and intensity of these infections, and (iii) assess the relative effect of* S. haematobium*,* P. falciparum*, and their coinfection on anaemia prevalence and severity in pregnancy.

## 2. Methods

### 2.1. Study Design

Malaria and urogenital schistosomiasis are coendemic in some areas of Mount Cameroon Area, Southwestern Cameroon. Munyenge is a rural community situated at the foot of mount Cameroon at an altitude of 261 above sea level. This village is endemic foci for the transmission of* S. haematobium* [23] and recent reports of a community-based study show an overall prevalence of 40% [32]. In the Mount Cameroon Area, malaria parasite transmission is perennial [33] and* P. falciparum* accounts for 60% of malaria parasite infection among pregnant women [34]. A cross-sectional study aimed to determine the prevalence of* S. haematobium*,* P. falciparum,* and coinfections and evaluate their relative effect on anaemia prevalence and severity among pregnant women is justified.

A sample size of 263 pregnant was determined to be adequate to detect a 5% change in prevalence. Sample size calculation was based on the estimate of the prevalence of* S. haematobium* infection in Munyenge according to a baseline epidemiological survey carried out in 2012 by Ntonifor et al. [23]. The sample size was determined using the formula n = z^2^pq/d^2^ [35], where n is the sample size required, z = 1.96 is confidence level test statistic at the desired level of significance, p = 78% is the proportion of urogenital schistosomiasis prevalence, q = 1 − p is the proportion of urogenital schistosomiasis negative, and d is the acceptable error willing to be committed. However, due to logistics, we had a sample size of 250 pregnant women which is well within 90–95% of the expected sample size calculated.

**Figure 1:**
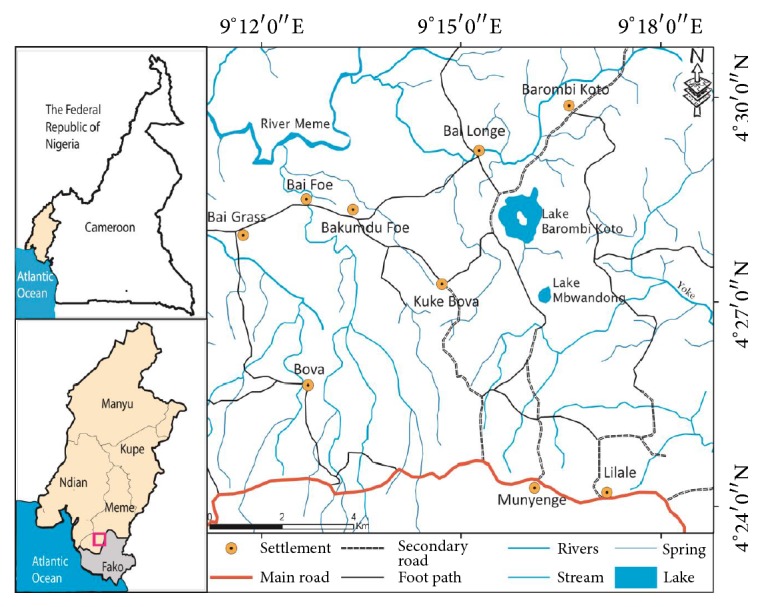
Map showing the location of Munyenge in Mount Cameroon Area.

The study was carried out in the Munyenge Health Area which is about 27 km from Muyuka town. It is bounded to the West by Likoko native, to the East by Masone, to the North by Mount Cameroon, and to the North East by Mbonge subdivision (Figure 1). Munyenge has a heterogenous population of 15,000 inhabitants consisting of individuals from several cultural backgrounds including natives from Oroko, Wimbum, kom, Mettas, Ibus, Nhies, Ndop, and Isimbis. This area is found in the rain forest of the Southwest Region with rich volcanic soil encouraging farming activities. The main occupation of the people is farming, with cocoa and plantains being their main cash crops [32]. Munyenge Health Area has four streams with outlet springs (providing natural water sources) situated in the middle of the village. These springs are habitats for the* Bulinus* spp. intermediate host and thus constitute the main transmission foci of* S. haematobium* in the community.* Bulinus* snails are found on vegetation and rocks surrounding the water point and on vegetation within the streams. These springs include the “coast timber” and “KCB” (men and women). The coast timber is a small catchment for spring water located within the community where pupils play often while the KCB “man” and “woman” as the name suggests are bathing springs for males and females, respectively, which are found further from the community. Munyenge is characterised by the absence of pipe borne water and the use of fresh water sources for household activities is common. All members of the community use these springs for drinking water, bathing, washing of clothes, and household utensils [32]. Munyenge has a temperature range of 24°C–27°C which favours high release of cercariae into the waters. These conditions make it certain that the people will continue to be infected and reinfected.

## 3. Data Collection

Pregnant women reporting for ANC clinic visit at the Munyenge Health Centre between June and September 2014, who had lived in Munyenge for at least two months and gave their consent, were enrolled consecutively. Pregnant women were interviewed by a field researcher using a questionnaire which recorded: demographic information (age, residence), gynaecologic/obstetric history (gravidity status, gestational age, and pregnancy complications), socioeconomic indicators (educational level, occupation), and questions related to malaria (IPTp-SP uptake, ITN usage) and schistosomiasis (main household water source, frequency of contact with open water source, and domestic activities carried out in the stream). In addition to the questionnaire interview, women were asked to collect urine in supplied 20 mL screw top plastic containers between 10:00 am and 2:00 pm. The samples were stored in a cool box during transportation to the University of Buea, Medical Research Laboratory, and processed within 24 hours of collection.

### 3.1. Laboratory Analyses

#### 3.1.1. Anaemia Status Determination

Haemoglobin levels were determined from finger-prick blood samples using a portable battery-operated photometer (HemoCue®) (HemoCue 201+ system, HemoCue, Angelholm, Sweden) (URIT-12). Hb concentration was expressed in g/dL. Anaemia was defined as an Hb value < 11.0 g/dL [36]. Anaemia severity was defined as follows: mild anaemia (Hb: 10–10.9 g/dL), moderate anaemia (Hb: 7–9.9 g/dL), and severe anaemia (Hb < 7.0 g/dL) [36].

#### 3.1.2. Parasitological Examination

Malaria parasites were identified on thick and thin blood smears stained with 5% Giemsa. The smears were observed for 30 minutes under the ×100 (oil immersion) objective of a UNICO® light microscope [37]. Malaria parasite density was estimated by counting parasites against 200 leucocytes in thick smears, assuming a white cell count of 8000 leucocytes per *μ*L of blood [38]. Malaria parasite density was classified into <500, 501–5,000, and >5,000 for low, moderate, and high parasitaemia, respectively [38].* S. haematobium* eggs were identified in urine samples using the filtration technique [39]. In brief, 10 mls of urine was filtered using membrane filters (Sterlitech Polycarbonate (PCTE) membrane filters, USA) and the egg count was recorded per 10 mls of urine. The infection intensity was classified as light (<50 eggs/10 mL of urine) or heavy (≥50 eggs/10 mL of urine) as defined by the World Health Organization (WHO) [40]. Microhaematuria was used as proxy-diagnosis of urogenital schistosomiasis, an accepted marker in the rapid diagnosis of* S. haematobium* infection in urine [41]. Samples were tested for microhaematuria using urine reagent strips (Uripath, Plasmatec Laboratory, UK) (Combi-11) as per manufacturer instructions. Results were expressed as negative or in levels of positivity (+, ++, or +++) and not including traces. A pregnant woman was infected with* S. haematobium* when she was diagnosed positive by microscopic examination and/or urine reagent strip.

### 3.2. Statistical Analysis

The data was analyzed using SPSS version 19.0 (SPSS, Inc., Chicago, IL, USA). Proportions of* S. haematobium*,* P. falciparum,* and coinfection and anaemia status were compared between different groups (age groups, gravidity status, educational level, occupational status, stream usage and activities, and IPTp-SP uptake) using Pearson Chi-square test. Crude odds ratios were estimated and factors associated with infections and anaemia to be included in the multivariate logistic regression model were identified. Variables that had a *P* value < 0.20 in bivariate analysis were included in the multivariate logistic regression model. Using the enter method, variables that showed independent association with infections and anaemia status at a significance level of *P* < 0.05 were retained in the model. Mean Hb levels were compared between groups using Analysis of Variance test (ANOVA) and Student's* t*-test. Independent factors associated with Hb levels were obtained using multilinear regression analysis. *P* values < 0.05 were considered significant.

### 3.3. Ethical Considerations

The study protocol and design including the consent procedures were approved by the Institutional Ethics Review Board of the Faculty of Health Science, University of Buea, and the Buea Regional Delegation of Public Health. Prior to conducting the study, the aim of the study and procedures to be used to collect data were explained to the pregnant women at the ANC clinic. Written (from those who can read and write) or verbal (from those who cannot read and write) informed consent from all study participants was obtained. Each pregnant woman who agreed to participate in the study was enrolled and given urine sample container to collect urine. Participation was voluntary and study participants were assured of confidentiality and anonymity of data.

## 4. Results

### 4.1. Characteristics of the Study Population

In this cross-sectional study, a total of 250 pregnant women reporting for antenatal care was enrolled. The mean age of the study participants was 25 ± 5.21 years (range: 14–40 years). The majority (75.2%) of the women were married. All the study participants had at least a primary education and about 50% had obtained some form of secondary education. Although a higher proportion of the women had been enrolled for first ANC in the second trimester, more than 40% had late clinic registration. About two-thirds of women reported taking at least one dose of IPTp-SP and 46% reported having slept under a bed net the previous night. The stream was the main source of water (99% stream usage) for domestic use and bathing. The characteristics of the study sample are shown in Table 1.

### 4.2. Prevalence and Intensity of Infection

#### 4.2.1. *S. haematobium* Infection

Of the 250 volunteer pregnant women enrolled, 117 (46.8%; 95% CI: 41–53) were positive for* S. haematobium* infection among whom 53 (45.3%) had heavy (≥50 eggs/10 mL of urine) infection while 54.7% (64) had light (<50 eggs/10 mL of urine) infection. The prevalence of microhaematuria was 9.6% (24/250). Using microscopic urine examination as gold standard, the specificity and sensitivity of microhaematuria in the diagnosis of* S. haematobium* infection were 100% (95% CI: 97.2–100) and 20.5% (95% CI: 14.2–28.7), respectively. Microhaematuria was strongly related to egg density categories where microhaematuria was common (*χ*^2^ = 8.23; *P* = 0.004) among women with heavy egg load (72.7%) than in those with light infection (27.3%).

#### 4.2.2. *P. falciparum* Infection

The overall prevalence of* P. falciparum* parasitaemia among the study participants was 39.2% (98) (95% CI: 33.4–45.4). Of the 98 pregnant women infected with* P. falciparum*, the proportions of low (<500 parasites/*μ*L of blood), moderate (501–5000), and high (>5000) parasitaemia were 43.9% (43), 52.0% (61), and 4.1 (4), respectively. About 15% (38) (95% CI: 11.3–20.2) of the pregnant women carried concurrent infections with* S. haematobium* and* P. falciparum*. Seventy-nine (31.6%) and sixty (24%) women had single infection with* S. haematobium* and* P. falciparum,* respectively.

#### 4.2.3. Factors Associated with Prevalence and Intensity of* S. haematobium* Infection

*S. haematobium* infection was associated with age, gravidity status, water contact frequency, type of activity carried out in the stream, and* P. falciparum* parasitaemia in bivariate analysis (Table 2). The prevalence of infection did not differ significantly with marital status, educational level, and occupational status. In multivariate analysis (controlling for age and gravidity status as confounders), younger age groups, ≤20 (aOR = 15.2 95% CI: 1.7–138.3) and 21–25 years (aOR = 7.3; 95% CI: 1.2–44.3), and bathing and domestic contact with stream (aOR = 33.5; 95% CI 9.7–115.9) were risk factors associated with* S. haematobium* infection. On the other hand, less water contact frequency (1 to 2 times per day) (aOR = 2.8*E* − 10; 95% CI: 9.4*E* − 11–8.5*E* − 10) was associated with decreased risk of infection. Surprisingly, primigravidity (OR = 0.2; 95% CI: 0.03–0.9) and secundigravidity (OR = 0.1; 95% CI 0.02–0.4) were less likely at risk. Intensity of infection was associated with malaria parasitaemia where light egg density infection was less common (aOR = 0.4; 95% CI: 0.2–0.7; *P* = 0.004) in malaria positive women (21.9%; 14/64) than in malaria negative women (78.1%; 50/64).

#### 4.2.4. Factors Associated with Prevalence of* P. falciparum* Infection

The prevalence of* P. falciparum* infection was associated (*χ*^2^ = 17.82; *P* < 0.01) with IPTp-SP uptake where malaria parasite infection was greater in women who had not taken IPTp-SP (58.0%) than in those who had at least one SP dose (30.2%). The occurrence of* P. falciparum* infection did not differ significantly with maternal age, gravidity status, or ITN usage.

#### 4.2.5. Factors Associated with Prevalence of Coinfection with* S. haematobium* and* P. falciparum*

Coinfection was associated with the type of activity carried out in the stream and water contact frequency as well as IPTp-SP uptake. Bathing and domestic contact with stream (aOR = 13.3; 95% CI 2.2–79.5) increased risk of coinfection among pregnant women; meanwhile, less water contact frequency (1 to 2 times per day (aOR = 0.1; 95% CI: 0.01–0.4) and 3 to 4 times per day (aOR = 0.3; 95% CI: 0.1–0.9)) decreased risk of coinfection. Women who had at least one SP dose were less likely (aOR = 0.06; 95% CI: 0.02–0.2) to be coinfected (Table 3).

### 4.3. Haemoglobin Levels and Anaemia

The mean (±SD) haemoglobin level of the pregnant women enrolled in the study was 9.0 ± 1.6 g/dL (range: 6.1–13.7 g/dL). Coinfection significantly reduced Hb levels of pregnant women in the study area where levels in coinfected individuals were significantly lower (*P* < 0.001) when compared with levels seen with single infections (*S. haematobium* and* P. falciparum*) and no infection (Table 4). In addition, Hb levels were significantly lower among women coinfected with* P. falciparum* and heavy* S. haematobium* infections than in individuals coinfected with* P. falciparum* and light* S. haematobium* infection and those with no infection (Table 5). Although age, marital status, educational level, occupational status, infection status, and IPTp-SP uptake were identified as factors associated with Hb levels, IPTp-SP was seen as the only independent predictor of Hb levels taking into consideration all possible confounding variables (Table 4).

Anaemia prevalence was 88.8% (222/250) with anaemia severity as follows: mild (13.2%; *n* = 33), moderate (62.8%; *n* = 157), and severe (12.8%; *n* = 32). All cases diagnosed with coinfection were anaemic (Table 6). Coinfection accounted for 93.8% (30/32) of all severe anaemia cases with majority 71.9% (23/32) of the severe anaemic cases coinfected with* P. falciparum* and heavy density* S. haematobium* infections (Table 5). Uptake of IPTp-SP (43.8%; 14/32) was associated (*χ*^2^ = 11.32; *P* = 0.01) with reduced percentage of severe anaemia compared with that observed among women with no SP (56.3%; 18/32). Risk factors found to be associated with increased odds of anaemia were* P. falciparum* infection (OR = 4.0, 95% CI: 1.0–14.5) and occupation (business) (OR = 20.1, 95% CI: 4.0–101) (Table 6).

## 5. Discussion

To our knowledge, this is the first study carried out on urogenital schistosomiasis among pregnant women in Cameroon. This study determined the prevalence of* S. haematobium*,* P. falciparum,* and coinfection, factors associated with these infections and assessed their relative effect on anaemia prevalence and severity among pregnant women in Munyenge.* S. haematobium* and* P. falciparum* infections are common among pregnant women living in Munyenge and their coinfection exacerbates anaemia.

The prevalence of* S. haematobium* infection among pregnant women in our study was 46.8%. The high prevalence reflects high exposure to infection among pregnant women living in Munyenge due to absolute dependence on natural water sources for domestic activities and bathing. Compared to the level of infection in the present study, lower prevalence of urogenital schistosomiasis among pregnant women has been reported in Nigeria by Eyo et al. [42] (23.8%) and Salawu and Odaibo [43] (20.8%). Differences in the method used for the detection of* S. haematobium* infection may partly explain the observed differences in rates. Although these studies attributed the lower prevalence levels of urinary schistosomiasis among pregnant women to a taboo restricting pregnant women from visiting natural water bodies [42], compared with urine filtration method used in our study, the lower sensitive centrifugation method use in the diagnosis of* S. haematobium* infection in the Nigerian studies may have underestimated true infection levels. Malaria is common among pregnant women in the study area with a prevalence of 39.2%. The only factor seen to be associated with malaria parasite infection in this study site was IPTp uptake. The effectiveness of IPTp-SP in the prevention of malaria in pregnancy is well established [34, 44].

For transmission of schistosomiasis to take place, the schistosomes parasite requires an avenue where it is in direct contact with the human host [9]. Pregnant women living in Munyenge get in contact with infection during activities such as laundry, plate washing, and water fetching for domestic use. In addition to domestic activities, bathing in streams poses a greater risk of infection among pregnant women in this area. Analyses from other studies have shown that regularly bathing in water sources contaminated with the developmental stages of the schistosomes parasite was associated with prevalence and intensity of schistosomiasis [45–47]. Moreover, increased risk of infection associated with the number and duration of water contact with infested waters per day has also been reported [47]. Women who reported surface-water contact at least 3 to 5 times per day were at greater risk of infection due to longer period of contact with contaminated water. Health education to instruct pregnant women to make less surface-water contact frequency and the implication of voiding their bladder in water bodies is paramount. These behavioural changes will significantly reduce the risk of* S. haematobium* infection among pregnant women and contamination of water sources in this setting. Ultimately, provision of portable water and improved sanitation system will play a major role in decreasing disease transmission and incidence.

Age, as observed in most schistosomiasis surveys, was a major determinant of schistosomes infection among pregnant women in our study area. The highest prevalence values of urogenital schistosomiasis were recorded in younger women (≤25 years). Individuals within ≤20 age group were found to be at a greater risk of* S. haematobium* infection with prevalence of 55.4%. This is in agreement with trends established in schistosomiasis surveys carried out in Cameroon [32, 48] and other parts of Africa [42, 43]. Alternatively, the decrease risk of infection observed in older age groups (>25 years) conformed to earlier reports [32, 42]. Studies have reported that age-acquired immunity to reinfection and changes in water contact patterns contribute to the declining trend in prevalence with increasing age [49]. Older women are less likely to be engaged in water contact behaviours compared to younger women. Age dependent immunity to* S. haematobium* has been shown to affect mean egg output of infected persons [49]. Socioeconomic status of the women was not an independent factor associated with* S. haematobium* prevalence in this high-risk community. Similarly, reports from other rural settings endemic for schistosomiasis failed to identify any socioeconomic variables that are strongly associated with schistosomiasis prevalence [50, 51]. The absence of association between socioeconomic variables and infection prevalence may be attributed to general poverty and uniformity in high exposure risk in the population [50].

The overall prevalence of coinfection with* S. haematobium* and* P. falciparum* infection was 15.2% suggesting coendemicity of both infections in the study area. Similarly, Yatich et al. [52] reported a helminth and malaria coinfection prevalence of 16.6% among pregnant women in Ghana. The impact of helminth infections on malaria parasitaemia and disease during coinfection is an established phenomenon although much is still unknown and contradictions persist [53, 54]. We observed that light* S. haematobium* infection was less common (aOR = 0.4) among pregnant women coinfected with* P. falciparum* suggesting a negative interaction between both parasites [4]. In accordance with findings of Getie et al. [55], schistosomiasis coinfection could affect* Plasmodium* parasitemia and vice versa, depending on the intensity of the ova in coinfected persons. Nonetheless, a further study is needed to explore the underlying mechanisms of interaction between malaria parasitaemia and* S. haematobium*.

Schistosomiasis causes long term morbidity such as anaemia. Our study showed that the magnitude of* S. haematobium* egg counts is significantly related to haemoglobin concentration confirming that urogenital schistosomiasis contributes to anaemia [24, 25]. In this study, anaemia was more pronounced in women with heavy infection intensity than in those with light infection. Coinfection of helminth infections and* P. falciparum* increases anaemia severity [8, 9]. Coinfection among pregnant women lowers Hb concentration compared with single infection. This is in agreement with findings of Okafor and Elenwo [56]. More so, coinfected women with heavy intensity* S. haematobium* infection had the lowest mean Hb levels (6.6 g/dL) and this subpopulation of women contributed to about 72% of all severe anaemic cases. The combined presence and interaction of* S. haematobium* and* P. falciparum* infections is partly responsible for the low haemoglobin concentration in women with concurrent infection. Malaria causes anaemia by destruction and removal of parasitized red blood cells and shortening of the life span of nonparasitized red cells as well as decreasing the rate of erythrocyte production in bone marrow [57]. The mechanism by which schistosomiasis causes anaemia is not fully understood but it is suggested that helminth infections could contribute to increase in the prevalence of inflammatory syndromes impairing erythropoiesis and interfering with mobilization of reticuloendothelial iron storages and shortening erythrocyte survival [58]. Similar to previous reports of a study in Uganda [59], malaria parasite infection was an independent factor associated with increase anaemia risk.

The risk of coinfection was associated with stream usage (bathing and domestic contact with stream) while less water contact and SP usage decreased risk of infection. This finding suggests that intervention strategies focusing on combating malaria and schistosomiasis, respectively, by increasing the uptake of IPTp-SP/doses and less water contact among pregnant women living in Munyenge represents the most appropriate prevention of coinfection with consequent increase in Hb levels.

This study had one limitation. We did not investigate the prevalence of HIV infection among the study participants. It has been shown that coinfections with helminths and malaria cause considerable morbidity in the host particularly in the presence of HIV infection [60].

To conclude, the study has indicated that* S. haematobium* and* P. falciparum* infections are common among pregnant women living in Munyenge and their coinfection is influenced by high frequencies of these parasites in the same population. The study also revealed that younger age and bathing and domestic contact with stream are independently associated with prevalence of* S. haematobium* infection while no IPTp-SP was associated with* P. falciparum* infection. Stream usage increased risk of coinfection while less water contact and SP usage decreased its risk. The fact that light* S. haematobium* infection was less common in* P. falciparum* infected women suggests that* Plasmodium falciparum* parasitaemia may be associated with intensity of urogenital schistosomiasis in coinfected individuals. Anaemia is a severe public health problem in pregnancy in Munyenge and coinfection with* S. haematobium* and* P. falciparum* exacerbates anaemia. Less water contact frequency and increase uptake of IPTp-SP/doses will significantly reduce risk of coinfection and consequently anaemia severity in pregnancy in this setting.

## Figures and Tables

**Table 1:** Characteristics of the study participants.

Characteristics	Number examined (*N*)	(%)
Age group (years)		
≤20	56	22.4
21–25	79	31.6
26–30	71	28.4
>30	44	17.6

Marital status		
Single	62	24.8
Married	188	75.2

Gravidity		
Primigravidae	69	27.6
Secundigravidae	71	28.4
Multigravidae	110	44.0

Trimester of first ANC		
First	10	4.0
Second	125	50.0
Third	115	46.0

Educational level		
Primary	123	49.2
Secondary	127	50.8

Occupation		
House wife	52	20.8
Business	93	35.2
Farmer	72	28.8
Student	33	13.2

Stream usage		
Yes	248	99.2
No	2	0.8

Activities in the stream		
Domestic contact and bathing	126	50.4
Domestic contact only	124	49.6

Frequency to streams/day		
1 to 2 times	122	48.8
3 to 4 times	57	22.8
5+ times	71	28.4

IPTp-SP uptake		
Yes	169	67.6
No	81	32.4

ITN use		
Yes	115	46.0
No	135	54.0

**Table 2:** Risk factors associated with *S. haematobium* infection among pregnant women in Munyenge.

Factors	Category	*S. haematobium* positive% (*n*)	Unadjusted OR (95% CI)	^#^Adjusted OR (95% CI)	*P* value
Age (years)	≤20	55.4 (31)	3.0 (1.3–6.8)	15.2 (1.7–138.3)	0.016
	21–25	53.2 (42)	2.7 (1.2–5.9)	7.3 (1.2–44.3)	0.031
	26–30	43.7 (31)	1.9 (0.8–4.1)	1.1 (0.2–6.1)	0.885
	>30	29.5 (13)	REF	REF	
	*χ* ^2^; *P* value	8.48; 0.037			

Gravidity	Primigravidity	63.8 (44)	2.7 (1.6–5.5)	0.2 (0.03–0.9)	0.034
	Secundigravidity	45.1 (32)	1.4 (0.6–2.5)	0.1 (0.02–0.4)	0.001
	Multigravidity	37.3 (41)	REF	REF	
	*χ* ^2^; *P* value	12.08; 0.002			

Marital status	Single	48.4 (30)	1.1 (0.6–1.9)	NA	
	Married	46.3 (87)	REF		
	*χ* ^2^; *P* value	0.83; 0.773			

Educational level	Primary	49.6 (61)	1.3 (0.8–2.1)	NA	NA
	Secondary	44.1 (56)	REF		
	*χ* ^2^; *P* value	0.74; 0.384			

Occupation	Housewife	42.3 (22)	1.3 (0.6–2.7)	2.5 (0.4–15.7)	0.333
	Business	52.7 (49)	1.8 (1.1–3.7)	4.3 (0.9–21.9)	0.079
	Student	60.6 (20)	2.7 (1.2–6.4)	1.6 (0.3–9.0)	0.587
	Farmer	36.1 (26)	REF	REF	
	*χ* ^2^; *P* value	7.55; 0.056			

Activities in the stream	Domestic contact and bathing	84.1 (106)	49.2 (22.4−107.7)	33.5 (9.7–115.9)	<0.001
	Domestic contact only	8.9 (11)	REF	REF	
	*χ* ^2^; *P* value	142.16; <0.001			

Frequency to the stream/day	1 to 2 times	13.1 (16)	0.14 (0.07–0.3)	2.8*E *− 10 (9.4*E* − 11–8.5*E* − 10)	<0.001
	3 to 4 times	52.6 (30)	REF	6.7*E *− 10 (6.7*E* − 10–6.7*E* − 10)	
	5+ times	100 (71)	—	REF	
	*χ* ^2^; *P* value	137.09; <0.001			

Malaria parasitaemia	Positive	38.8 (38)	0.6 (0.4–1.0)	0.4 (0.14–1.2)	0.098
	Negative	52 (79)	REF	REF	
	*χ* ^2^; *P* value	4.17; 0.041			

*χ* ^2^: Pearson Chi-square test; OR: odd ratio.

^#^OR adjusted using multivariate regression analysis.

**Table 3:** Risk factors associated with coinfection with *S. haematobium* and *P. falciparum* among pregnant women in Munyenge.

Factors	Category	Presence of coinfection% (*n*)	Unadjusted OR (95% CI)	^#^Adjusted OR (95% CI)	*P* value
Age (years)	≤20	25 (14)	4.6 (1.2–17)	2.8 (0.3–22.7)	0.338
	21–25	17.7 (14)	2.9 (0.8–10.9)	1.5 (0.2–9.7)	0.662
	26–30	9.9 (7)	1.5 (0.4–6.1)	0.3 (0.04–2.2)	0.247
	>30	6.8 (3)	REF	REF	
	*χ* ^2^; *P* value	8.53; <0.001			

Gravidity	Primigravidity	23.6 (16)	3.4 (1.4–8.2)	0.4 (0.1–1.7)	0.198
	Secundigravidity	18.3 (13)	2.5 (1.0–6.2)	0.9 (0.2–3.5)	0.893
	Multigravidity	8.2 (9)	REF	REF	
	*χ* ^2^; *P* value	8.15; 0.017			

Marital status	Single	17.7 (11)	1.3 (0.6–2.8)	NA	
	Married	14.4 (27)	REF		
	*χ* ^2^; *P* value	0.41; 0.52			

Educational level	Primary Secondary *χ*^2^; *P* value	13.8 (17) 6.5 (31) 0.36; 0.55	1.4 (0.7–2.9) REF	NA	

Occupation	Housewife	13.5 (7)	1.2 (0.4–3.7)	NA	
	Business	17.2 (16)	2.1 (0.8–5.3)		
	Student	21.2 (7)	3.0 (1.0–9.2)		
	Farmer	11.1 (8)	REF		
	*χ* ^2^; *P* value	2.27; 0.518			

Activities in the stream	Domestic contact and bathing	26.6 (36)	24.4 (5.7–104)	13.3 (2.2–79.5)	0.005
	Domestic contact only	1.6 (2)	REF	REF	
	*χ* ^2^; *P* value	35.24; <0.001			

Frequency to the stream/day	1 to 2 times	2.5 (3)	0.04 (0.01–0.15)	0.1 (0.01–0.4)	0.002 0.027
	3 to 4 times	15.8 (9)	0.3 (0.1–0.8)	0.3 (0.1–0.9)	
	5+ times	36.6 (26)	REF	REF	
	*χ* ^2^; *P* value	40.65; <0.001			

IPTp-SP uptake	Yes	9.5 (16)	0.3 (0.1–0.6)	0.06 (0.02–0.2)	<0.001
	No	27.2 (22)	REF	REF	
	*χ* ^2^; *P* value	13.29; <0.001			

*χ* ^2^: Pearson Chi-square test; OR: odd ratio.

^#^Adjusted OR using multivariate regression analysis.

**Table 4:** Factors associated with mean (±SD) haemoglobin levels among pregnant women in Munyenge Health Area.

Factors	Category	Mean (±SD) Hb levels	Test-value	Unadjusted *P* value	*t*-test	^&^Adjusted *P* value
Age (years)	≤20	8.6 ± 1.7	^*∗*^ *F* = 3.35	0.02	2.02	0.045
	21–25	9.1 ± 1.7				
	26–30	9.0 ± 1.4				
	>30	9.5 ± 1.5				

Gravidity	Primigravidity	8.9 ± 1.9	*F* = 0.75	0.474	NA	NA
	Secundigravidity	8.9 ± 1.4				
	Multigravidity	9.1 ±1.5				

Marital status	Single	8.5 ± 1.4	^$^ *t* = −2.70	0.007	1.79	0.075
	Married	9.2 ± 1.6				

Educational level	Primary	9.3 ± 1.7	*t* = 2.42	0.016	−1.62	0.106
	Secondary	8.8 ± 1.5				

Occupation	Housewife	9.6 ± 1.8	*F* = 10.16	<0.001	0.19	0.852
	Business	8.7 ± 1.3				
	Student	8.0 ± 1.2				
	Farmer	9.4 ± 1.7				

Infection status	*S. haematobium* only	9.5 ± 1.6	*F* = 31.61	<0.001	−1.22	0.225
	*P. falciparum* only	9.1 ± 1.2				
	Coinfection	7.0 ± 1.0				
	No infection	9.5 ± 1.4				

IPTp-SP uptake	Yes	9.2 ± 1.6	*t* = 2.95	0.004	−2.60	0.01
	No	8.6 ± 1.5				

^*∗*^Analysis of variance test (ANOVA).

^$^Student's *t*-test.

^&^Adjusted *P* values using multilinear regression analysis.

NA: not applicable: variables with *P* > 0.2 in bivariate analysis were not included in regression analysis.

**Table 5:** Association between *S. haematobium* intensity, *P. falciparum* infection, and mean (±SD) haemoglobin levels and anaemia severity.

*S. haematobium *egg intensity	*P. falciparum *infection status	*N*	Mean (±SD) Hb levels	Anaemia severity (% (*n*))			
				Mild	Moderate	Severe	^&^Significance level
Light	Positive	14	7.8 ± 1.2	0 (0)	50 (7)	50 (7)	*χ* ^2^ = 29.71; *P* < 0.001
	Negative	50	9.4 ± 1.7	14 (7)	72 (36)	0 (0)	

Heavy	Positive	24	6.6 ± 0.3	0 (0)	4.2 (1)	95.8 (23)	*χ* ^2^ = 41.72; *P* < 0.001
	Negative	29	9.3 ± 1.3	6.9 (2)	72.4 (21)	6.9 (2)	

Negative	Positive	60	8.3 ± 1.5	16.7 (10)	76.7 (46)	0 (0)	*χ* ^2^ = 4.2; *P* = 0.122
	Negative	73	9.5 ± 1.5	19.2 (14)	63.0 (46)	0 (0)	

^*∗*^Significance level	*F* = 12.40; *P* < 0.001						

^*∗*^Analysis of variance test (ANOVA).

^&^Pearson Chi-Square test.

**Table 6:** Risk factors associated with anaemia among pregnant women in Munyenge Health Area.

Factors	Category	Anaemia prevalence	^#^Adjusted OR (95% CI)	*P* value
Age (years)	< or = 20	92.9 (52)	NA	
	21–25	87.3 (69)		
	26–30	90.1 (64)		
	>30	84.1 (37)		
	*χ* ^2^; *P* value	2.21; .531		

Gravidity	Primigravidity	87 (60)	NA	
	Secundigravidity	94.4 (67)		
	Multigravidity	86.4 (95)		
	*χ* ^2^; *P* value	3.10; 0.212		

Marital status	Single	93.5 (58)	0.8 (0.2–3.2)	0.79
	Married	87.2 (164)	REF	
	*χ* ^2^; *P* value	1.87; 0.17		

Educational level	Primary	80.5 (99)	0.1 (0.04–0.5)	0.001
	Secondary	96.9 (123)	REF	
	*χ* ^2^; *P* value	16.82; <0.001		

Occupational status	Housewife	80.8 (42)	1.5 (0.5–4.0)	0.458
	Business	97.8 (91)	20.1 (4.0–101)	0.001
	Student	100 (33)	6.3*E*8 (6.3*E*8–6.3*E*8)	—
	Farmer	77.8 (56)	REF	
	*χ* ^2^; *P* value	23.99; <0.001		

*P. falciparum* infection status	Positive	95.9 (94)	4.0 (1.1–14.5)	0.037
	Negative	84.2 (128)	REF	
	*χ* ^2^; *P* value	8.21; 0.004		

IPTp-SP uptake	Yes	86.4 (146)	1.1 (0.3–3.8)	0.866
	No	93.8 (76)	REF	
	*χ* ^2^; *P* value	3.05; 081		

*S. haematobium* infection status	Positive	90.6 (106)	NA	
	Negative	87.2 (116)		
	*χ* ^2^; *P* value	0.71; 0.40		

Coinfection status	Presence	100 (38)	14.5*E*8 (0.0 - )	0.998
	Absence	86.8 (184)	REF	
	*χ* ^2^; *P*-value	5.65; 0.017		

^#^Adjusted OR using multivariate regression analysis.

NA: not applicable: variables with *P* > 0.2 in bivariate analysis were not included in multivariate analysis.

## Abbreviations

IPTp-SP: Intermittent preventive treatment in pregnancy (IPTp) with sulfadoxine-pyrimethamine (SP)
ITN: Insecticide treated bed nets
HIV: Human immunodeficiency virus
ANC: Antenatal care.
